# Microbial Communities Associated with the Larval Gut and Eggs of the Western Corn Rootworm

**DOI:** 10.1371/journal.pone.0044685

**Published:** 2012-10-02

**Authors:** Flavia Dematheis, Benedikt Kurtz, Stefan Vidal, Kornelia Smalla

**Affiliations:** 1 Institute for Epidemiology and Pathogen Diagnostics, Julius Kühn-Institut - Federal Research Centre for Cultivated Plants (JKI), Braunschweig, Germany; 2 Department of Crop Science, Agricultural Entomology, Georg-August Universität Göttingen, Göttingen, Germany; University of Waterloo, Canada

## Abstract

**Background:**

The western corn rootworm (WCR) is one of the economically most important pests of maize. A better understanding of microbial communities associated with guts and eggs of the WCR is required in order to develop new pest control strategies, and to assess the potential role of the WCR in the dissemination of microorganisms, e.g., mycotoxin-producing fungi.

**Methodology/Principal Findings:**

Total community (TC) DNA was extracted from maize rhizosphere, WCR eggs, and guts of larvae feeding on maize roots grown in three different soil types. Denaturing gradient gel electrophoresis (DGGE) and sequencing of 16S rRNA gene and ITS fragments, PCR-amplified from TC DNA, were used to investigate the fungal and bacterial communities, respectively. Microorganisms in the WCR gut were not influenced by the soil type. Dominant fungal populations in the gut were affiliated to *Fusarium* spp., while *Wolbachia* was the most abundant bacterial genus. Identical ribosomal sequences from gut and egg samples confirmed a transovarial transmission of *Wolbachia* sp. Betaproteobacterial DGGE indicated a stable association of *Herbaspirillum* sp. with the WCR gut. Dominant egg-associated microorganisms were the bacterium *Wolbachia* sp. and the fungus *Mortierella gamsii.*

**Conclusion/Significance:**

The soil type-independent composition of the microbial communities in the WCR gut and the dominance of only a few microbial populations suggested either a highly selective environment in the gut lumen or a high abundance of intracellular microorganisms in the gut epithelium. The dominance of *Fusarium* species in the guts indicated WCR larvae as vectors of mycotoxin-producing fungi. The stable association of *Herbaspirillum* sp. with WCR gut systems and the absence of corresponding sequences in WCR eggs suggested that this bacterium was postnatally acquired from the environment. The present study provided new insights into the microbial communities associated with larval guts and eggs of the WCR. However, their biological role remains to be explored.

## Introduction

The western corn rootworm (WCR), *Diabrotica virgifera virgifera* LeConte (Coleoptera: Chrysomelidae), is one of the economically most important pests of maize *(Zea mays* L.) in the US and it is an increasing threat to corn-growing areas in Europe [1]. In the US the WCR causes about $1.3 billion in lost revenue and control costs each year [2], while in Europe potential damage costs of € 470 million per year are expected [3]. Major yield losses are caused by WCR larvae feeding on root tissues resulting in reduced growth and plant lodging. The high adaptability of this herbivorous insect to prevailing pest management strategies such as annual crop rotation with soybean [1], [4] or WCR-resistant transgenic plants [5] alerted maize farmers worldwide. Thus, new pest control strategies are required, and in this respect microorganisms intimately associated with the gut of insects are an emerging research topic as they might be used as targets of new pest control measures [6], [7], [8], [9]. During the root feeding, the WCR larvae can ingest soil-borne plant pathogens which can remain viable after passage through the gut system [10]. Therefore, a deeper insight into the microbiome associated with the gut of the WCR might also help to predict the spreading of phytopathogenic microorganisms or mycotoxin producing fungi through WCR larvae feeding on maize roots.

Microorganisms inhabiting the digestive tracts of insects can play important roles in the nutrition, development, survival, resistance to pathogens, and reproduction of the insect host [11], [12], [13], [14], [15], [16]. Furthermore, Broderick et al. [17] showed that gut bacteria are required for *Bacillus thuringiensis* insecticidal activity. Bacteria, intracellularly located in the gut epithelium or present in the lumen of the gut system [18], [19], [20], can be vertically acquired from the parent by transovarial transmission [21], [7], [22], capsule transmission (deposition of bacterium-containing capsules with eggs) [14], [23], and egg smearing (superficial egg contamination) [24]. The microbial composition of the gut of insects is assumed to depend also on external parameters such as dietary and environmental factors [25], [26], [27], [28].

Despite the general importance of the microorganisms inhabiting the digestive tract, little is known about the microbial composition in the WCR gut. Some studies reported the presence of symbiotic *Wolbachia* strains in the WCR gut [29], while only Molnár et al. [30] investigated the yeast diversity in the gut of the WCR. However, no studies on bacterial and fungal communities in the gut of the WCR, and on their transovarial transmission were reported until now. Furthermore, no studies elucidated the egg-associated microbiome of this pest. Recently, feeding of WCR larvae was shown to alter the relative abundance of bacterial communities in the rhizosphere of maize [31]. Because the larvae are feeding on maize roots with tightly adhering soil, we hypothesized that different soil-borne microorganisms can be ingested, influencing the microbial composition in the digestive tract of WCR larvae. So far, no studies have been published on the influence of different soil types on the microbial communities in the gut of soil dwelling insects.

The main objectives of this work were (i) to investigate the effect of the soil type on the composition of microbial communities inhabiting the digestive tract of the WCR, (ii) to identify the most dominant gut-associated microorganisms; (iii) to investigate their transovarial transmission or their putative origin from the rhizosphere, and (iv) to identify the dominant microbial populations associated with WCR eggs. To achieve these goals internal transcribed spacers (ITS) and 16S rRNA gene fragments amplified from TC DNA extracted from samples of gut and eggs of the WCR, and maize rhizosphere were analyzed by denaturing gradient gel electrophoresis (DGGE). Dominant microorganisms harboring gut and eggs of the WCR were identified by cloning and sequencing of ITS and 16S rRNA gene fragments.

## Materials and Methods

### Experimental Setup

Maize plants were grown in the greenhouse in pots (13 cm diameter) containing three different soil types: Haplic Chernozem, Haplic Luvisol and Eutric Vertisol. Gauze was glued to the bottom of these pots to prevent the escape of the larvae. Four independent replicates per soil type were prepared for pots with WCR larvae and controls. Sixty eggs of WCR were injected directly into the soil close to the plant stems three weeks after sowing (growth stage V3). The plants were grown for another three weeks. After 21 days of larval feeding on the maize roots, the larvae (3^rd^ instars) were collected from the soil (see below) and their gut was immediately removed for the total communities (TC) DNA extraction. In parallel, the plants (growth stage V7) were harvested for the TC DNA extraction from rhizosphere soil.

### Soil Types and Sampling Method

Three different soil types, Haplic Chernozem, Eutric Vertisol and Haplic Luvisol, were collected nearby Göttingen (Germany) in 2008 from the upper 25 cm, each from four spots five meters apart from each other along a transect. Physico-chemical parameters (e.g., pH, particle size, nitrogen and carbon content) and microbial composition differed among soil types as shown in a previous work [31]. In order to avoid any alteration of the microbial content, the soil samples were immediately transported to the laboratory and used for the experiments after homogenization using a soil crusher machine (Unifix 300, Möschle, Ortenberg, Germany) and sieving through a 10 mm mesh.

### WCR Egg Source, Stimulation of the Larval Development and Hatch Test

Non-diapausing WCR eggs were provided by USDA-ARS (Northern Grain Insect Research Laboratory, Brookings, SD, USA) and stored at 8°C until their use. In order to stimulate the larval development, the eggs were incubated at 26°C, 60% relative humidity in dark conditions for 12 days and checked for visible larval presence using a dissecting microscope. Afterwards the eggs were washed in a sieve (250 µm diameter) and the collected eggs were suspended in 0.15% agar solution. 0.5 mL of egg suspension were applied on a sterile humid filter paper and incubated at the same conditions as described for larval development and checked daily to assess the hatch time (HT) and the hatch rate (HR). The HT and HR mean values were two days and 72%, respectively. No specific permits were required for the described greenhouse studies.

### Maize Variety and Growing Conditions

The maize variety used in this study was KWS 13, an early maturing Northern European flint x dent maize breeding line developed by the seed company KWS (Einbeck, Germany). The maize growing conditions adopted in our experiments were the following: 40% relative humidity, 24°C mean temperature and 16 h of additional illumination with sodium lamps (400W, HS2000, Hortilux Schréder, Monster, The Netherlands). Plants grown in the same soil were placed within the same tray that was moved twice a week in the greenhouse to randomize the growing conditions. Fertilizer Hakaphos blau (Compo, Münster, Germany; 2.5%) was applied by watering once a week to plants older than 14 days.

### Extraction of WCR Larvae from the Soil and Gut Isolation

After 20 days of feeding, the larvae were extracted from the soil by means of a high gradient Kempson extraction system [32]. The larvae were washed three times with sterile double-distilled H_2_O and sedated with ethanol (40%). Afterwards, the larvae were cut at both ends and the gut was removed aseptically using a tweezer. Single and composite gut samples were prepared. For the composite samples ten guts of larvae grown in the same pot were pooled to obtain approximately 25 mg fresh weight.

### WCR Egg Surface Sterilization and Conservation

The WCR eggs were washed in a sieve (200 µm diameter) with cold water and transferred to 30 mL of a 5% MgSO_4_ solution for about 1 min. The material that sank was transferred into 65% MgSO_4_ solution. Eggs floating to the surface were sampled and washed with tap water. Subsequently the eggs were transferred into 2 mL reaction tubes containing a sterile washing solution consisting of 0.85% NaCl and 0.1% Tween, and vortexed for 30 s. The eggs were transferred to a Petri dish containing sterile water and placed under UV light for one night. The eggs were dried on sterile filter paper and transferred to a solution of 0.33 g Nipagin per ml of 70% ethanol. After 30 min the eggs were washed and stored in 70% ethanol. The efficiency of the surface sterilization was checked twice plating 50 eggs on Potato Dextrose Agar (Merck). No microbial growth was observed in the following two weeks.

### Microbial DNA Extraction from Rhizosphere, Gut and Egg Samples

Plants were vigorously shaken and the soil tightly adhering to the roots was considered as rhizosphere. The rhizosphere was collected by a Stomacher blender (Stomacher 400, Seward, UK) following the method described by Costa et al. [33]. The microbial pellet was obtained from the cell suspensions by centrifugation at 10.000 *g* at 4°C for 30 min. The microbial pellet of each root was homogenized with a spatula and 0.5 g was used for the TC DNA extraction with the FastDNA SPIN Kit for Soil (Q-Biogene, Carlsbad, CA, USA) according to the manufacturer’s instructions. Because the microbial fingerprints of individual guts were highly variable (Supplemental information, Fig. S2), TC DNA was extracted from composite samples of ten guts. TC DNA from pools of ten guts and from four pools of 100 surface sterilized eggs were extracted using the same kit as for the rhizosphere DNA extraction following the manufacturer’s protocol with some modifications: the material was placed into bead tubes, frozen in liquid nitrogen and subsequently processed for 1 min at speed 5.5 m s^−1^ in a FastPrep system (Bio-101, Vista, CA, USA); the TC DNA pellet was eluted in 100 µL of TRIS-EDTA buffer (pH 7.4) included in the kit. All TC DNA samples were purified with the GENECLEAN Spin Kit (Q-Biogene, Heidelberg, Germany) according to the manufactureŕs protocol. DNA concentrations were estimated visually by 0.8% agarose gel electrophoresis using the quantitative marker High DNA Mass Ladder (Invitrogen). TC DNA from rhizosphere and from eggs was diluted 1∶10 for PCR amplifications, while undiluted TC DNA from guts was used as a PCR template.

### PCR Amplification of the 18S and 16S rRNA Gene Fragments and ITS Fragments for DGGE Analysis

The 18S rRNA gene fragments of the fungal communities from gut samples were amplified by a semi-nested PCR amplification. The primer pair NS1 and EF3 was used in the first PCR reaction, while NS1 and FR1-GC were used in the second amplification. Reaction mixture and PCR conditions were described by Oros-Sichler et al. [34]. The ITS fragments of the fungal communities from gut and egg samples were amplified using a nested PCR approach with the primer pair ITS1F/ITS4 and ITS 2/ITS1F-GC according to Weinert et al. [35]. The 16S rRNA gene fragments of complex bacterial communities were amplified by direct PCR performed with the primer pair F984GC/R1378 as described by Heuer et al. [36]. The amplification of the 16S rRNA gene fragments of *Pseudomonas*, *Alphaproteobacteria*, *Betaproteobacteria,* and *Actinobacteria* was carried out with taxon specific primers in a nested PCR amplification according to Weinert et al. [35].

### DGGE, Cluster Analysis and Statistics

18S rRNA gene fragments amplified from TC DNA were analyzed in the DCode™ System (Biorad Laboratory, Hercules, CA, USA) as described by Oros-Sichler et al. [34]. ITS- and 16S rRNA gene fragments were analyzed in DGGE gels run in the PhorU2 machine (Ingeny, Goes, The Netherlands) according to Weinert et al. [35]. Gels were silver stained and air dried according to Heuer et al. [37]. Gel images were digitally captured using an Epson 1680 Pro scanner (Seiko-Epson, Japan) with high resolution setting. Digitalized DGGE gel images were analyzed with the software package GELCOMPAR II program, version 4.5 (Applied Math, Kortrijk, Belgium) as described by Rademaker et al. [38]. Background was subtracted and lanes were normalized as described by Gomes et al. [39]. Cluster analysis (UPGMA) based on pairwise sample similarity was performed. A permutation test was applied on pairwise similarities of community fingerprints according to Kropf et al. [40] to evaluate if the differences observed were statistically supported. P values <0.05 indicate significant differences between treatments.

### ITS Clone Library and Screening on DGGE Gel

Products of the first ITS amplification with primers ITS1F/ITS4 (circa 600 bp) obtained from TC DNA of gut or egg samples were cloned using the pGEM-T vector system (Promega). ITS inserts of positive transformants were re-amplified by PCR using the primer pair ITS 1F-GC/ITS2 and re-analyzed by DGGE to check their electrophoretic mobility. For gut samples, five to nine clones per soil type carrying the insert representative for the most dominant fungal population were selected for sequencing. For each egg sample, five clones carrying ITS fragments with different DGGE electrophoretic mobility were sequenced.

### Identification of Bands of the Bacterial DGGE Gels

Dominant bands (i.e. thicker bands) were excised from DGGE gels. The gel slices were transferred to a 1.5 mL tube and crushed with the top of a sterile tip. DNA was eluted from the gel slices by incubation overnight at 4°C in sterile TE buffer at pH 8. After centrifugation at 11,000×*g* for 60 s, the supernatant was transferred to a new tube and 1 µL was used as template for the reamplification. PCR products were cloned using the pGEM-T vector system (Promega). Cloned fragments were amplified with the primers F984-GC/R1378 and analyzed by DGGE for correspondence with specific DGGE bands. Four to six clones per excised DGGE band were sequenced. ITS sequences were first analyzed by BLAST-n searches in GenBank at the NCBI site. The 16S rRNA gene sequences were analyzed by CLASSIFIER at RDP (Ribosomal Database Project) to identify the sequences at the genus level, and with BLAST-n searches in GenBank. ITS and 16S rRNA sequences obtained from gut and egg samples were aligned using Clustal W in MEGA 4.0 software. Phylogenetic trees were constructed with MEGA 4.0 using the neighbor-joining algorithm and 500 repetitions for the calculation of the bootstrap values.

#### Nucleotide sequence accession numbers

Nucleotide sequences determined in this study were deposited in the GenBank database under accession numbers JF461095-JF461251 (Table 1 and 2).

**Table 1:** Fungal species identified in the larval gut and/or in the eggs of the WCR, accession numbers of ITS sequences obtained by cloning of specific DGGE bands from gut and egg fingerprints, and bands source.

Fungal species and ITS sequence identity (ID)	Gut	Eggs	Band
*Candida sake* (AJ549822), 99% ID	JF461105, −115	**–**	band a (Fig. 1)
*Gibberella zeae* (AB250414), 100% ID	JF461098, −9, −101, −102, −108, −110, −112, −115	**–**	band d (Fig. 1)
*Verticillium dahliae* (DQ282123), 97% ID	JF461104	**–**	band e (Fig. 1)
*Fusarium* spp. (EU750680; EU750688), 98% ID	JF461095, −6, −7, −103, −106, −109, −110, −111, −113, −114, 116, −107	**–**	band c (Fig. 1)
*Fusarium* spp. (FJ460589;EU750687), 99% ID	**–**	FJ461124, −7, −9, −39	band 2 (Fig. 2)
*Mortierella gamsii* (DQ093723), 98% ID	**–**	JF461117, −23, −25, −26, −28, −30, −33, −35, −38, −40 to −55, −57, −60, −61,−64, −65, 67, −69, −71 to −95	band 1 (Fig. 2)
*Cylindrocarpon olidum* (AJ677294), 98% ID	**–**	JF461134, −56, −58, −59, −62, −63	band 3 (Fig. 2)
*Trichocladium asperum* (AM292050), 100% ID	**–**	JF461166 and JF461170	band 4 (Fig. 2)

**Table 2:** Bacterial species identified in the larval gut and/or in the eggs of the WCR, accession numbers of 16S gene fragment sequences obtained by cloning of specific DGGE bands from gut and egg fingerprints, and bands source.

Bacterial species and sequence identity (ID)	Gut	Eggs	Band
*Wolbachia* (AY007551), 99–100% ID	JF461205, −7, −8	JF461210	band 1 (Fig. 5A)
*Wolbachia* (AY007551), 99–100% ID	JF461204, −6, −9	JF461211	band 2 (Fig. 5B)
*Duganella* sp. (EF592558), 99–100% ID	***–***	FJ461212, −5, −8	band 3 (Fig. 5C)
*Herbaspirillum* sp. (EU341291), 98% ID	JF461196 to −200, −202, −203	***–***	band 3 (Fig. 5C)
bacterium endosymbiont of *Mortierella elongata* (AB558492), 96% ID	***–***	JF461213, −14, −16, −17, −19, −20	band 4 (Fig. 5C)
*Pseudomonas* sp. (GU377209; EU118771; EU834404),99–100% ID	JF461248 to −51	JF461237, −38, −40, −41, −43 to −45	band 5 (Fig. 5D)
*Azotobacter chroococcum* (AB696772), 99% ID	***–***	JF461240	band 5 (Fig. 5D)
*Lysobacter* sp. (AB299978; DQ191178; FN600120), 99% ID	***–***	JF461239, −42, −46, −47	band 6 (Fig. 5D)
*Streptomyces* sp. (EF37143; CP002475), 100% ID	JF461232 and JF461233	JF461221 to −5, −7 to −9	band 7 (Fig. 5E)
*Rhodococcus* sp. (AB458522; AM497794), 99–100% ID	***–***	JF461226 and JF461230	band 8 (Fig. 5E)
*Tsukamurella sp.* (AB564289), 98–100% ID	JF46131, −4 to −6	***–***	band 8 (Fig. 5E)

## Results

### Fungal Communities in the Gut of WCR Larvae

**Figure 1:**
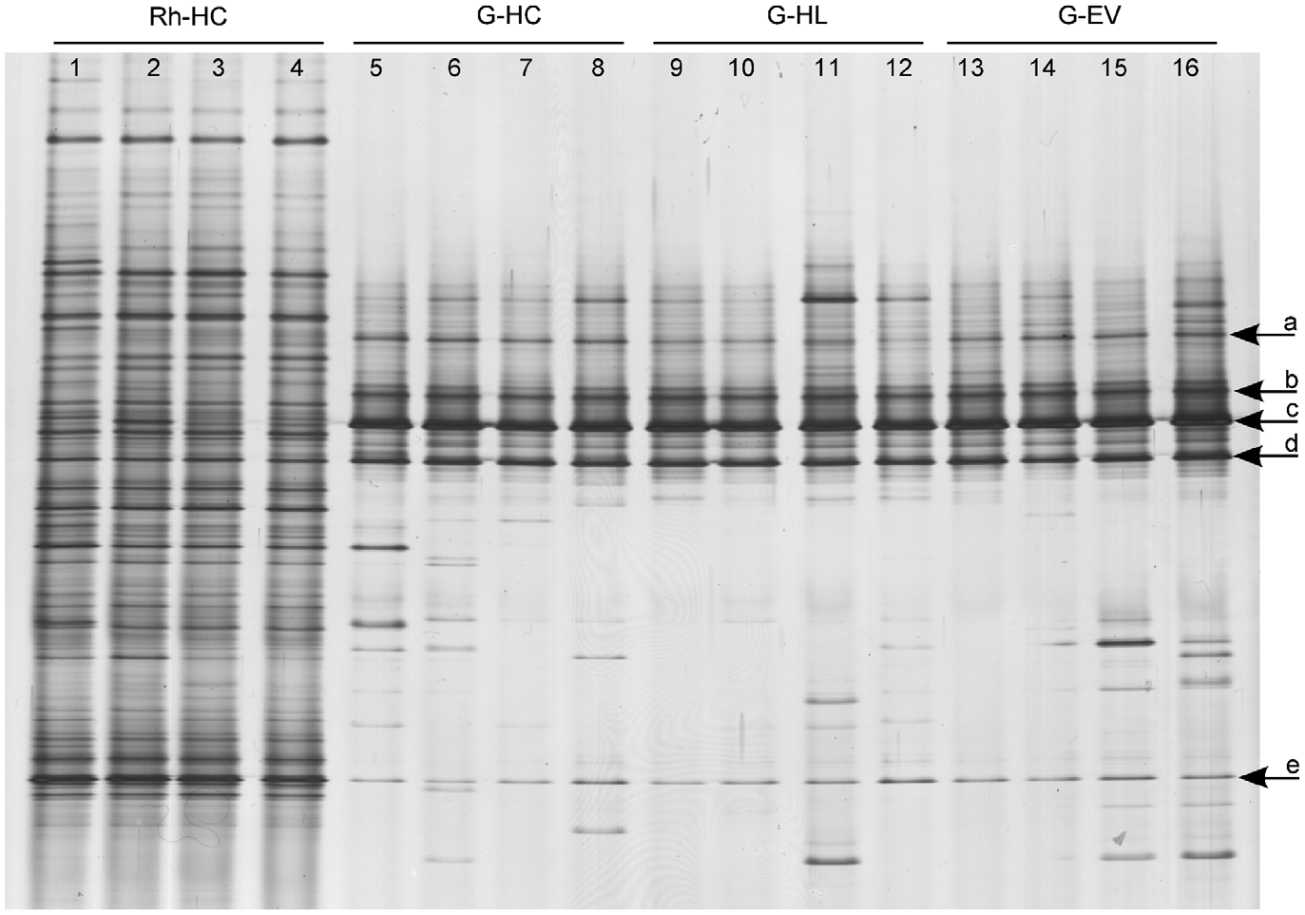
Fungal DGGE profiles showing the fungal community structure in the rhizosphere of maize plants grown in Haplic Chernozem (Rh-HC) and in the gut of WCR larvae feeding on maize roots grown in Haplic Chernozem (G-HC), in Haplic Luvisol (G-HL) and in Eutric Vertisol (G-EV). Arrows indicate dominant fungal populations identified by sequencing (Table 1). Band a: *Candida sake*; band c: *Fusarium* sp.; band d: *Gibberella zeae*; band e: *Verticillium dahliae*.

In order to identify the most appropriate molecular marker for typing the fungal communities associated with the gut of the WCR, ITS and SSU (18S) rRNA DGGE fingerprints were compared. DGGE of ITS fragments showed more complex band patterns than those of 18S rRNA gene fragments, which displayed only one dominant band for all gut samples and few faint bands (Supplemental Information, Fig. S1). Therefore, ITS-based DGGE analysis was chosen to investigate the fungal communities associated with WCR intestines. To investigate the influence of the soil type on gut-associated fungi, ITS fragments amplified from TC DNA extracted from guts of larvae sampled in three different soil types were analyzed by DGGE fingerprinting. Five dominant bands (a–e) were detected in all fingerprints of guts independently from the soil types (Fig. 1). In contrast to the complex fungal fingerprints of maize rhizosphere samples, the DGGE patterns of WCR guts showed a strongly reduced number of bands. Cluster analysis confirmed that the fungal communities in the gut of larvae grown in the different soil types were highly similar, sharing more than 80% similarity (data not shown). Statistical analysis based on the Pearson correlation indices confirmed that the composition of WCR gut-associated fungi was not significantly affected by the soil type.

### Identification of Fungi in the Guts of WCR Larvae

The most dominant fungi associated with the gut of WCR larvae grown in the three different soil types were identified by cloning and sequencing of ITS1F/ITS4 PCR products. Most of the cloned ITS fragments showed the same electrophoretic mobility as band c (68.3%) and band d (17.3%) (Fig. 1). Only two cloned inserts co-migrated with band a and only one with band e. None of the cloned ITS fragments co-migrated with band b. The remaining clones carried inserts with electrophoretic mobilities not corresponding to dominant bands in the ITS-DGGE fingerprints. One to 12 clones per soil type carrying an insert co-migrating with bands a, c, d, and e (Fig. 1) were selected for sequencing (Table 1). Sequences of band a were affiliated to *Candida sake* (AJ549822) with 99% identity (ID), while ITS sequences of band c showed 98% ID with *Fusarium* spp. (EU750680; EU750688). Sequences of bands d and e were identified as *Gibberella zeae* (AB250414) and *Verticillium dahliae* (DQ282123) with 100% and 97% ID, respectively.

### Origin of the Dominant Fungi in WCR Larval Gut

**Figure 2:**
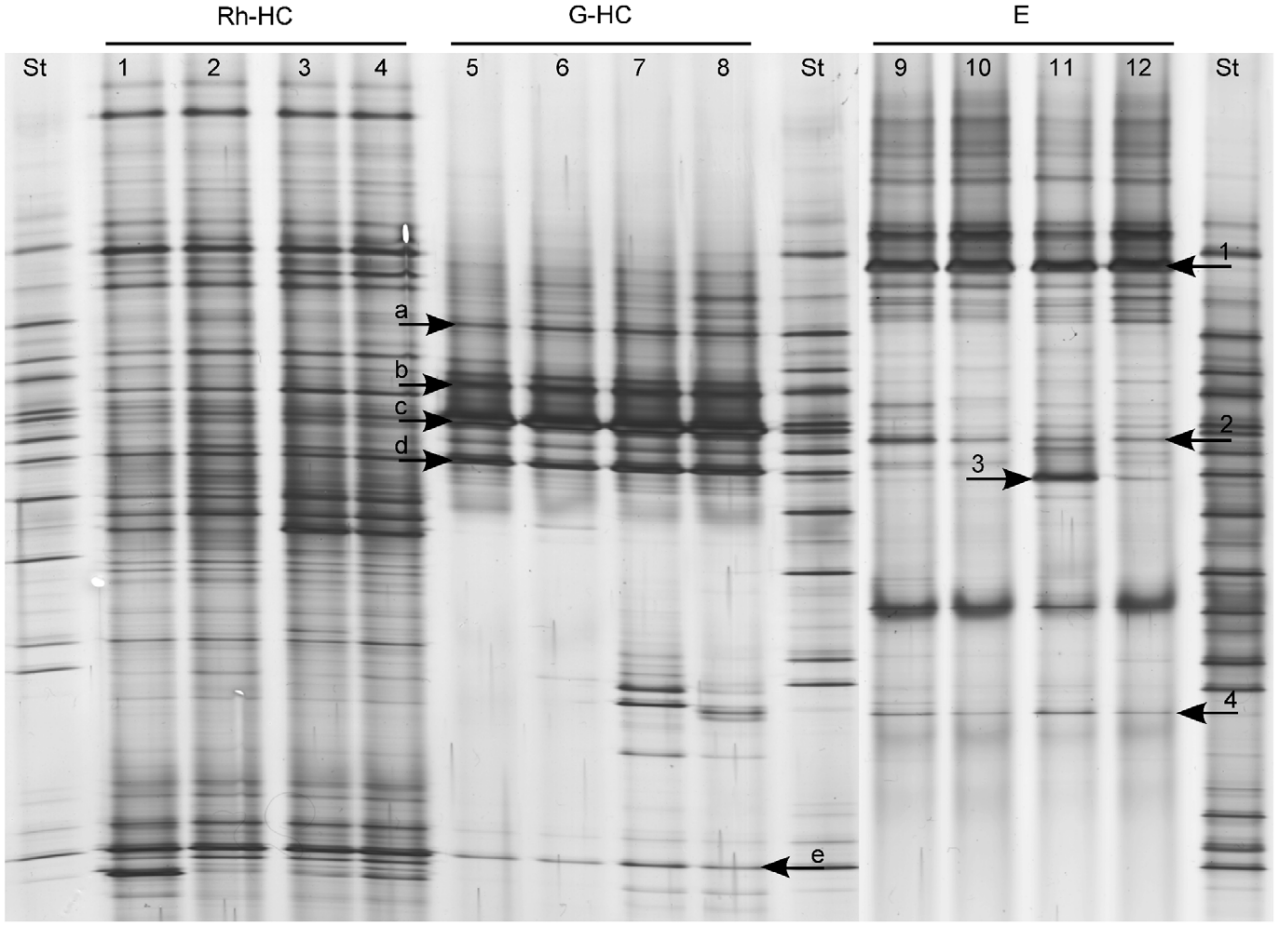
Bacterial DGGE fingerprints obtained from rhizosphere samples of maize plants grown in Haplic Chernozem (Rh-HC), gut samples of larvae collected from the soil Haplic Chernozem (G-HC) and surface sterilized egg samples (E). St: ITS standard. Arrows indicate bands for which cloned ITS fragments with the same electrophoretic mobilities were sequenced (Table 1). Band 1: *Mortierella gamsii*; band 2: *Fusarium* sp.; band 3: *Cylindrocarpon olidum*; band 4: *Thrichocladium asperum;* band a: *Candida sake*; band c: *Fusarium* sp.; band d: *Gibberella zeae*; band e: *Verticillium dahliae*.

**Figure 3:**
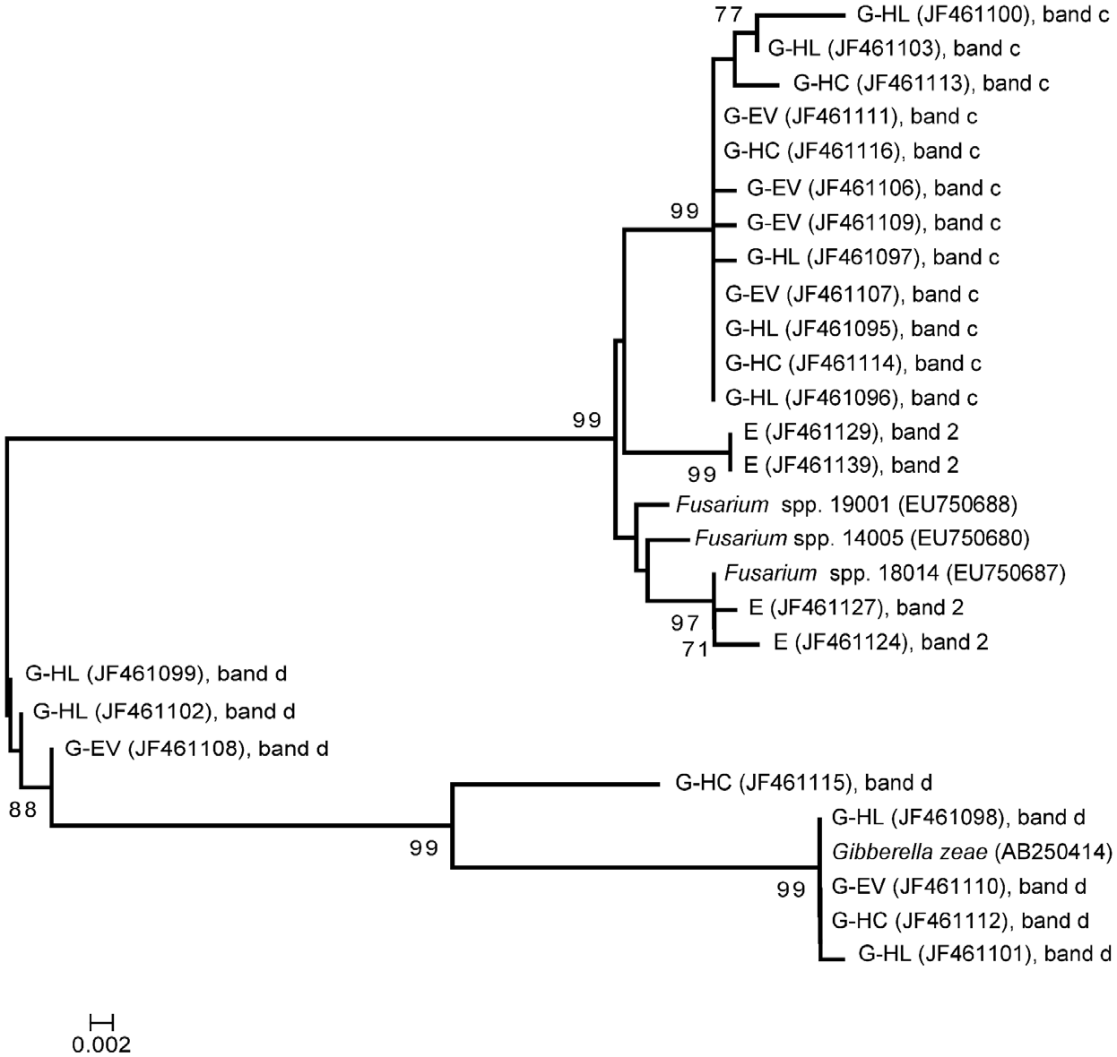
Neighbor-joining tree derived from ITS sequences amplified from TC DNA extracted from surface sterilized eggs (E) and gut of WCR larvae feeding on maize plants grown in Haplic Chernozem (G-HC), in Haplic Luvisol (G-HL) and in Eutric Vertisol (G-EV). Each sequence is labeled with the corresponding GenBank accession number, and the corresponding DGGE band in Fig. 1 and 2. The dendrogram was generated with MEGA 4 software. The branches show bootstrap values higher than 60%.

To investigate whether the most dominant fungi detected in the WCR gut were transovarially transmitted rather than taken up during the root larval feeding from the rhizosphere, fungal DGGE fingerprints of gut, rhizosphere and egg samples were compared (Fig. 2). Distinct fungal fingerprints were observed for each sample type. No congruence of any of the dominant bands was observed, except for the dominant band identified as *Verticillium dahliae* in the ITS-DGGE of gut (band e, Fig. 1) which occurred also in the fungal fingerprinting of the maize rhizosphere; and for band c in Fig. 2, identified as *Fusarium* spp. in the fingerprints of the gut, which was observed as a faint band in both rhizosphere and egg samples. ITS sequences of this band obtained from egg profiles revealed 99% ID with *Fusarium* spp. (FJ460589 and EU750687) as well. However, the phylogenetic analysis of all ITS sequences identified as *Fusarium* spp. obtained from gut and egg samples showed that *Fusarium* sequences from the gut clustered separately from those from eggs (Fig. 3).

### Identification of the Most Abundant Egg-associated Fungi

DGGE of the fungal communities associated to WCR eggs is reported in Fig. 2. In order to identify the dominant fungal populations associated with WCR eggs a clone library of the ITS1F/ITS4 PCR products from TC DNA of WCR eggs was generated. The majority (85%) of the cloned fragments had the same DGGE electrophoretic mobility of the dominant band (band 1). Sequence analysis showed that the most dominant fungal population in WCR eggs displayed 98% ID with *Mortierella gamsii* (DQ093723). The sequences of the faint band 2 from the WCR eggs shared 99% ID with *Fusarium* spp. (FJ460589; EU750687).The sequences obtained from bands 3 and 4 were affiliated to *Cylindrocarpon olidum* (AJ677294) with 98% ID and to *Trichocladium asperum* (AM292050) with 100% maximal identity, respectively (Table 1).

### Bacterial Community in the Gut of WCR Larvae

**Figure 4:**
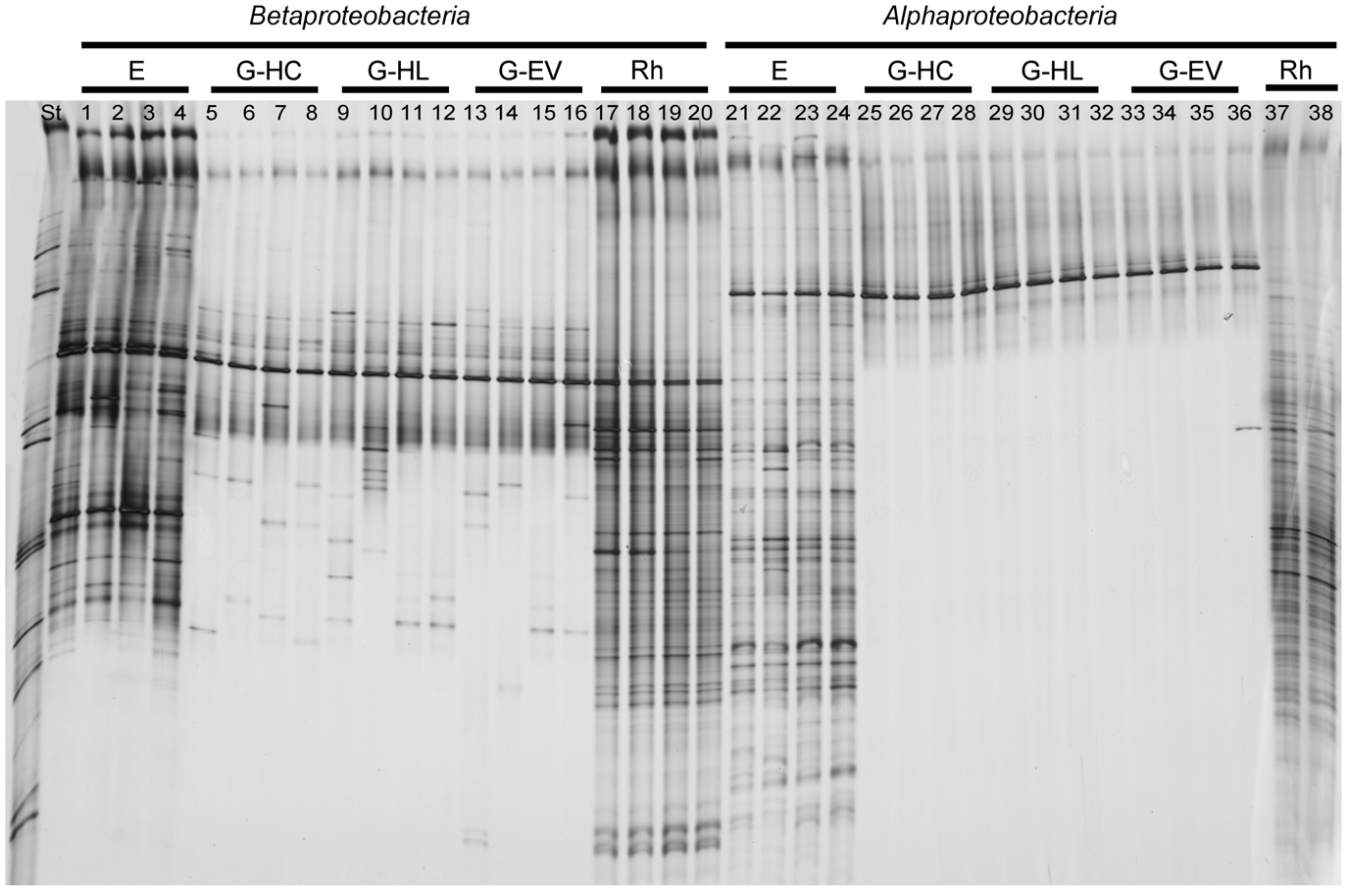
Alpha- and betaproteobacterial DGGE of surface sterilized eggs (E) and gut of WCR larvae grown in Haplic Chernozem (G-HC), in Haplic Luvisol (G-HL) and in Eutric Vertisol (G-EV). The fingerprinting of the alpha- and betaproteobacterial communities in the rhizosphere of maize grown in Haplic Chernozem (Rh) is reported as well. St: standard. The gel shows no soil type effect on the *Alpha-* and *Betaproteobacteria* in the digestive tract of WCR larvae.

The 16S rRNA gene fragments of total bacteria and of four different bacterial taxonomic groups (*Alphaproteobacteria, Betaproteobacteria*, *Pseudomonas,* and *Actinobacteria*) amplified from TC DNA were analyzed by DGGE in order to investigate the effect of the soil type on the bacterial composition inhabiting the gut of WCR larvae. Total bacterial and lphaproteobacterial DGGE profiles of the guts were very similar to each other (data shown only for *Alphaproteobacteria* in Fig. 4): both DGGE fingerprints showed highly similar patterns among replicates and gut samples of larvae collected in different soil types; only one dominant band with identical electrophoretic mobility was observed in all DGGE fingerprints of guts. Statistical comparison of the total bacterial and alphaproteobacterial fingerprints of WCR guts by means of the permutation test described by Kropf et al. [40] did not reveal a significant effect of the soil type on the gut microbiome. Similarly, also the betaproteobacterial DGGE showed just one dominant band with the same electrophoretic mobility in all replicates and gut samples independently from the soil type from where the larvae were collected (Fig. 4). Statistical analysis showed that the soil type did not influence the *Betaproteobacteria* in WCR guts. *Pseudomonas* and actinobacterial communities in the guts showed high variability among replicates in DGGE gels (data not shown). Thus, no evidence for an influence of the soil type was found.

### Identification of Gut-associated Bacteria in WCR Larvae

Sequencing of the dominant band in the fingerprints of the total bacterial communities showed a high similarity to *Wolbachia* sp. (AY007551) with 99% ID. The same species was identified by sequencing of the dominant band in the DGGE fingerprints of the *Alphaproteobacteria*. Sequencing of the dominant band in betaproteobacterial fingerprints revealed in all gut samples a bacterial population affiliated to *Herbaspirillum* sp. (EU341291) with 98% ID. *Wolbachia* sp. and *Herbaspirillum* sp. were identified in the gut of WCR larvae sampled in all three soil types. Because the fingerprints of *Pseudomonas* and *Actinobacteria* in WCR larval guts showed a high variability among replicates (no common populations in the gut were observed), no specific bands from those communities were investigated.

### Origin of Bacteria in WCR Larval Guts and Identification of Egg-associated Bacteria

**Figure 5:**
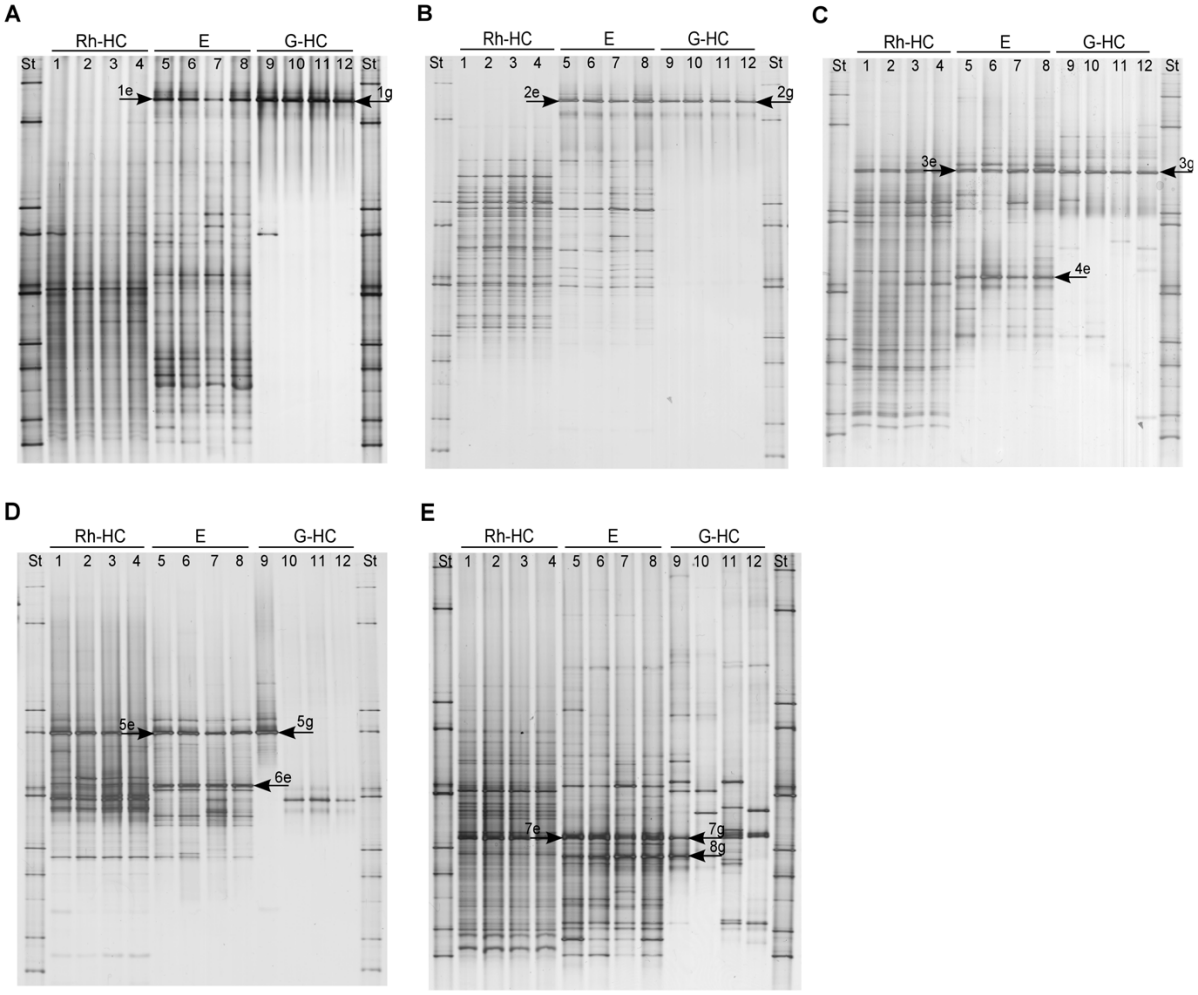
Bacterial DGGE fingerprints obtained from rhizosphere samples of maize plants grown in Haplic Chernozem (Rh-HC), gut samples of larvae collected from the soil Haplic Chernozem (G-HC) and surface sterilized egg samples (E). (A) Total bacterial DGGE; (B) Alphaproteobacterial DGGE; (C) Betaproteobacterial DGGE; (D) Pseudomonas DGGE, and (E) actinobacterial DGGE. Arrows indicate bands excised from the gels for sequencing (Table 2). Bands 1g and 1e, and band 2g and 2e: *Wolbachia* sp.; band 3g: *Herbaspirillum* sp.; band3e: *Duganella* sp.; band 4e: bacterial endosymbiont of *Mortierella elongata*; bands 5g and 5e: *Pseudomonas* sp.; band 6e: *Lysobacter* sp.; bands 7g and 7e: *Streptomyces* sp.; band 8g: *Tsukamurella* sp.; band 8e: *Rhodococcus* sp.

**Figure 6:**
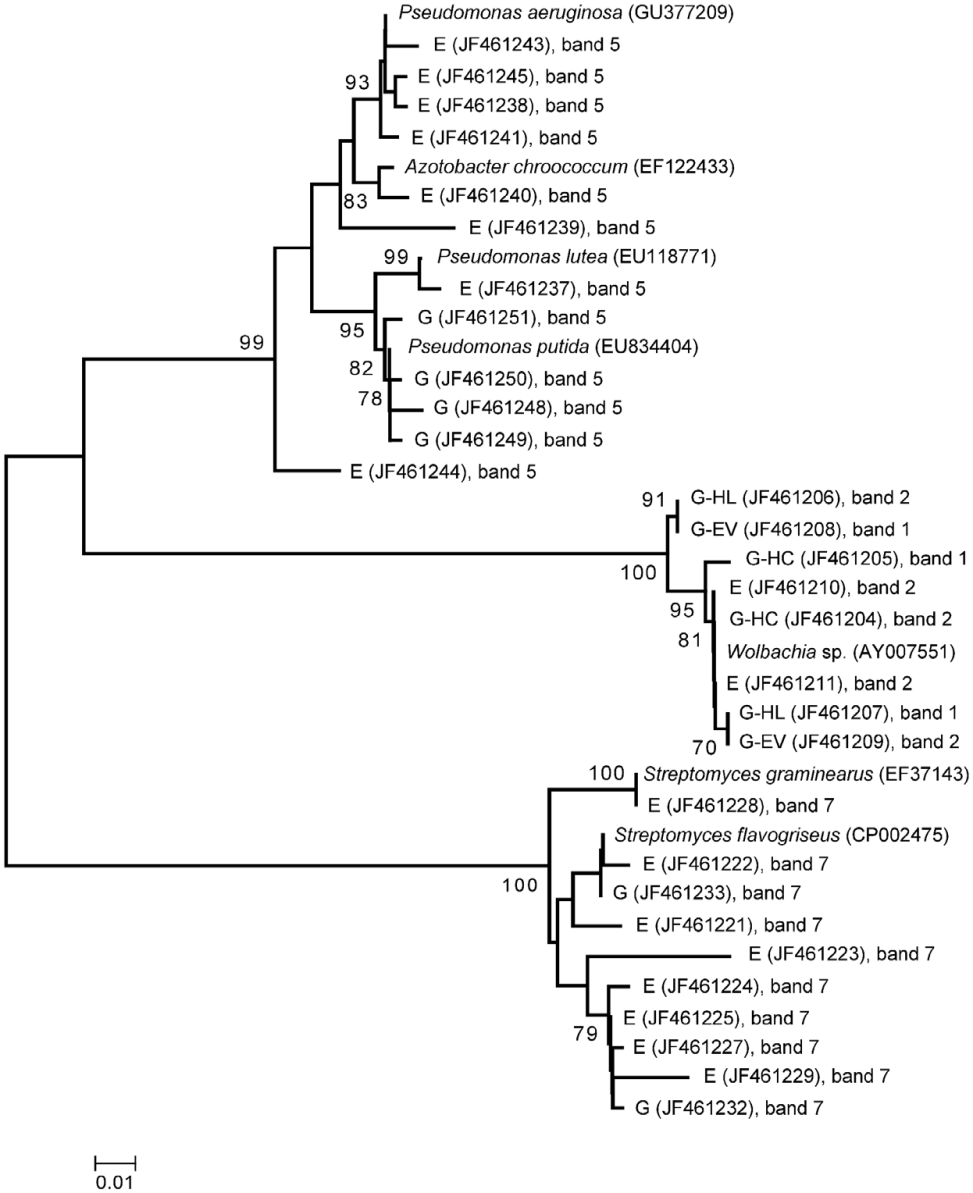
Neighbor-joining tree derived from 16S rRNA sequences isolated from surface sterilized eggs (E) and gut samples obtained from WCR larvae feeding on maize plants grown in Haplic Chernozem (G-HC), in Haplic Luvisol (G-HL) and in Eutric Vertisol (G-EV). Each sequence is additionally labeled with the corresponding GenBank accession number, and the bands source in Fig. 5. The dendrogram was generated with MEGA 4 software. The branches show bootstrap values higher than 60%.

In order to explore the potential origin of the gut-associated bacteria, the bacterial fingerprints of maize rhizosphere, WCR guts and eggs were compared. The egg fingerprints were more complex and consisted of several bands compared to the ones obtained from gut samples. The dominant band, identified as *Wolbachia* sp. in the bacterial and alphaproteobacterial fingerprints of guts (band 1g in Fig. 5A and band 2g in Fig. 5B), occurred in the DGGE fingerprints of eggs as well (band 1e in Fig. 5A and band 2e in Fig. 5B). Sequencing of this band and the phylogenetic analysis revealed in both eggs and guts identical *Wolbachia* sequences (Fig. 6). No band with the same electrophoretic mobility was detected in the rhizosphere fingerprints.

The betaproteobacterial DGGE (Fig. 5C) showed that the dominant band (band 3g) which was identified as *Herbaspirillum* sp. in the fingerprints of gut samples was also detected in the rhizosphere patterns. A band with slightly different electrophoretic mobility of band 3g was observed in the egg betaproteobacterial patterns (band 3e). Cloning and sequencing of this band revealed 99–100% sequence similarity to *Duganella* sp. (EF592558). The sequencing of a second dominant band in the betaproteobacterial DGGE patterns of the eggs (band 4e) displayed 96% ID with a bacterial endosymbiont of *Mortierella elongata* (AB558492). This band was neither detected in the gut nor the rhizosphere DGGE fingerprints.

*Pseudomonas* DGGE profiles (Fig. 5D) showed one band which was dominant and common among all rhizosphere and egg samples, and one replicate of the gut samples (band 5). The sequencing of band 5e excised from *Pseudomonas* DGGE fingerprint of the eggs revealed 99–100% sequence similarity to *Pseudomonas aeruginosa* (GU377209), *P. lutea* (EU1187719) and *Azotobacter chroococcum* (AB696772). The sequencing of the corresponding band in one of the replicates of the gut samples (band 5g) displayed 99% ID with the same *P. putida* (EU834404). Band 6e was observed only in the *Pseudomonas* DGGE fingerprint of eggs. The sequences obtained from band 6e were affiliated by sequencing to *Lysobacter spongiicola* (AB299978), *L. daejeonensis* (DQ191178), and to *L. gummosus* (FN600120) with 99% ID.

Actinobacterial DGGE profiles (Fig. 5E) showed one band which was dominant and common among all rhizosphere and egg samples, and few replicates of the gut samples (band 7). The sequences of band 7e excised from the WCR eggs fingerprint showed the highest sequence similarity to *Streptomyces graminearus* (EF37143) and *S. flavogriseus* (CP002475) with 100% ID. Sequences of a co-migrating band (band 7g) in one of the gut fingerprints were affiliated to *S. flavogriseus* as well. However, no identical sequences originating from eggs and guts were found. The actinobacterial DGGE showed in the eggs a second dominant band (band 8e) which occurred also in the fingerprints of some of the gut replicates (band 8g). Band 8e sequences were affiliated by sequencing to *Rhodococcus* sp. (AB458522; AM497794) with 99–100% ID, while band 8i sequences showed the highest sequence similarity to the genus *Tsukamurella* (AB564289) with 98–100% ID.

## Discussion

The present study provides new insight into the gut microbiome of WCR larvae and WCR egg-associated microorganisms. Analyses of gut samples were performed using TC DNA of the complete gut and therefore this work provides data on fungal and bacterial microorganisms present in both lumen and gut epithelium of WCR larvae. Compared to the microbial DGGE fingerprints of the maize rhizosphere, the corresponding fingerprints of the guts of WCR larvae displayed a strongly reduced complexity, indicating a highly selective condition in the digestive system. In contrast to the rhizosphere microbial communities of maize plants that were recently shown to be significantly influenced by the soil type [31], no soil type effect was observed on the composition of the gut microbiome of WCR larvae. Although Robinson et al. [41] showed that substantial temporal variation of the midgut bacterial communities can occur in cabbage white butterfly larvae, in this study no temporal changes of the microbial communities in the digestive tract of WCR were addressed, as the guts were obtained from 3^rd^ instars larvae feeding on maize roots of plants at V3 growth stage.

Dominant fungal populations associated with WCR guts were mainly affiliated to *Fusarium* spp. and *Gibberella zeae* which is the teleomorphic form of *F. graminearum*. Molnár et al. [30], investigating the yeast communities in the gut flora of WCR larvae by DGGE of the D1 domain of the 26S rRNA gene, identified these fungi as well. The unspecific amplification of *Fusarium* spp. and *Gibberella zeae* when using yeast-specific primers can likely be explained by their high relative abundance in the WCR guts. No indication of their numerical dominance in the digestive system of WCR was previously reported.

Comparative DGGE analyses of maize rhizosphere, WCR guts and eggs, followed by ITS sequence analyses of dominant populations allowed us to investigate the potential origin of these populations in the guts of WCR larvae. ITS sequences affiliated to *Gibberella zeae* were found in the guts but not in egg samples, suggesting that this fungal population was not transovarially transmitted. DGGE analysis revealed in the rhizosphere patterns a band with a similar electrophoretic mobility of the band identified as *Gibberella zeae* in the microbial fingerprints of the digestive tracts (Fig. 2), indicating a potential origin of this fungus from the rhizosphere. Unfortunately, we failed to clone this band from the rather complex rhizosphere patterns. ITS sequences affiliated to *Fusarium* sp. were found in both guts and egg samples. The phylogenetic analysis of these sequences revealed a different origin of *Fusarium* spp. found in the guts from that one in the eggs. The detection of *Fusarium* in the eggs could be caused by *Fusarium*-egg infection, while *Fusarium* in the guts could be originated from the rhizosphere, as the rhizosphere fingerprints showed a band with the same electrophoretic mobility of the band identified as *Fusarium* spp. in the microbial profiles of WCR guts. Because the WCR gut has a pH of 5.5 approximately [42], [43], [44], and the majority of the *Fusarium* species are tolerant to acid and alkaline pHs, it is reasonable to speculate that the conditions in the gut of WCR larvae selected these fungal populations. The plating of the homogenate of digestive tracts of WCR larvae on *Fusarium* selective media revealed *Fusarium* species (Kurtz et al., personal communication), indicating that the fungus remains viable in the gut of larvae. The finding that *Fusarium* spp. are dominant and viable in the digestive tract of the WCR clearly indicated that WCR larvae might be viewed as vectors of potentially mycotoxin-producing *Fusarium* species, corroborating results published by Palmer and Kommedahl [10]. This also explains the increased colonization of maize roots by *Fusarium verticilloides* observed in presence of WCR larval feeding [45].

Two other fungal populations affiliated to *Candida sake* and *Verticillium dahliae* showed a stable association with the guts of the WCR larvae. *C. sake* was one of the most frequent yeast species in WCR larval guts identified by Molnár et al. [30]. This suggested an important role of this yeast in the functional biology of the gut of the insect. Differently, no previous studies reported *V. dahliae* in the gut of the WCR. This soil-borne fungus is classified among parasites of vascular tissues of hops and several other dicotyledonous plants. Comparative DGGE analyses of maize rhizosphere and WCR guts indicated an external environmental origin of this microbial population. Due to the lack of data concerning the viability of *Verticillium dahliae* in the gut, we cannot speculate the dissemination of this phytopathogenic fungus via WCR larvae, as we did for *Fusarium* spp. However, this topic can be of interest for further studies.

DGGE fingerprints of the fungal population in the eggs revealed a pronounced band and several faint bands. The complex profile can be explained if we assume that the surface sterilization of the eggs did not totally exclude the DNA of surface-associated microorganisms. The major fungal population identified in the eggs shared 98% similarity with *Mortierella gamsii* (*Zygomycota*). The relative high abundance of this fungus suggested an important role in the WCR biology which might be a matter of further investigations.

DGGE fingerprints of total bacterial and alphaproteobacterial communities in the guts of WCR larvae displayed only one dominant band which was identified as *Wolbachia* sp. The low complexity of the DGGE profiles can be due to the high cell numbers of *Wolbachia* sp. population inside the epithelial cells of the WCR gut. The presence of identical 16S rRNA sequences of *Wolbachia* sp. identified in WCR guts and eggs confirmed the transovarial transmission of this bacterium to the offspring. The absence of a band with the electrophoretic mobility of *Wolbachia* sp. in the total bacterial and alphaproteobacterial DGGE of rhizosphere samples confirmed the already known obligate symbiotic relationship between WCR and *Wolbachia* sp. Several studies reported on the presence of *Wolbachia* sp. in *Diabrotica* spp. beetles, an intracellular bacterium maternally transmitted to the offspring and responsible for reproductive incompatibilities between infected and uninfected individuals [12], [46], [47]. Recently Barr et al. [29] showed that *Wolbachia* sp. colonizing the WCR insect is responsible for the down-regulation of the maize plant defences suggesting an important role of this microorganism in the pathogenicity of the insect.

DGGE analysis of group-specific bacteria and band sequencing allowed us to identify minor populations [36] which were not detectable in the total bacterial community fingerprints due to the numerical dominance of *Wolbachia* sp. Interestingly, also the betaproteobacterial DGGE fingerprints displayed a low complexity pattern with just one dominant band affiliated by sequencing to *Herbaspirillum* sp., and no soil type dependent differences. Several studies reported *Herbaspirillum* sp. as an intestinal microorganism of different insect species [48], [49]. Recently, *Herbaspirillum* sp. has also been identified as a secondary symbiont of a citrus psyllid [50]. The stable association of *Herbaspirillum* sp. with the digestive tract of WCR might indicate an intracellular location of this bacterium. The absence of *Herbaspirillum* sp. sequences in WCR eggs indicated either a low abundance of this microorganism compared to other species in the eggs (e.g. *Duganella* sp.), or a putative origin from the rhizosphere or plant roots. Previous studies reported *Herbaspirillum* sp. as nitrogen-fixing endophytes in rice and maize plants [51], [52].

In contrast to the alpha- or betaproteobacterial DGGE, the actinobacterial and *Pseudomanoas* fingerprints displayed a high degree of variability among replicates which might be caused either by a low abundance of these bacterial groups to be PCR-amplified or a transient association of these taxa with the guts of WCR larvae.

Several bacterial populations were reported here for the first time being associated to WCR eggs. Bacterial populations identified in the WCR eggs were *Wolbachia* sp., *Duganella* sp., and a betaproteobacterial population which showed 96% similarity with a bacterial endosymbiont of *Mortierella elongata*. The low sequence similarity of the latter population precluded its clear taxonomic identification, thus it may be assumed a novel species. However, the finding of *Mortierella gamsii* as a dominant egg-associated fungus suggested a multitrophic interaction among WCR insect, fungi and bacteria that might be of interest for forthcoming studies. Other dominant bacterial populations identified in WCR eggs belonged to the genus *Pseudomonas* sp., *Lysobacter* sp., *Streptomyces* sp., and *Rhodococcus* sp. Several studies reported these microorganisms in the intestine of earthworms [53], [54] and termites [55]. 16S rRNA gene sequences of *Streptomyces* and *Pseudomonas* were found also in few gut samples. However, because no identical sequences originating from eggs and guts were found, a transovarial transmission of these microbial populations is unlikely. *Lysobacter* sp. was amplified using specific primers for *Pseudomonas,* indicating that the primer specificity was reduced in presence of a high abundance of *Lysobacter* sp. The broad-spectrum of enzymes [56] and antibiotics inhibiting bacteria and fungi produced by *Lysobacter* sp. [57], [58], [59], [60] might suggest its potential activity in the guts of the WCR larvae.

Overall, the microbial communities of WCR eggs seemed to be more complex than those of the larval guts, and except for *Wolbachia* sp. no identical sequences were obtained from both sample types.

Although the present study provided exciting novel insights into the microbial communities associated with the gut of larvae and eggs of the WCR, their biological role remains to be investigated in view of identifying targets for new pest control strategies.

## Supporting Information

Figure S1 Fungal (18S rRNA gene) DGGE fingerprints obtained from single gut of WCR larvae grown in Haplic Chernozem (G-HC), in Haplic Luvisol (G-HL) and in Eutric Vertisol (G-EV). St: 18S-standard.
(TIF)

Figure S2 Fungal (ITS) DGGE fingerprints of the fungal communities in the rhizosphere of maize plants grown in Haplic Chernozem (Rh), in single gut samples obtained from WCR larvae feeding on maize plants grown in Haplic Chernozem (G-HC), and DGGE profiles of cloned ITS fragments from single gut samples (clones). Fungi identified by sequencing and blast analysis of cloned ITS fragments are reported above the corresponding DGGE band in the figure.
(TIF)
