# Supplementary figures and images for: Microbial Communities Associated with the Larval Gut and Eggs of the Western Corn Rootworm
Source: PLoS One. 2012 Oct 2;7(10):e44685. doi: 10.1371/journal.pone.0044685 (PMC3462784; doi:10.1371/journal.pone.0044685)

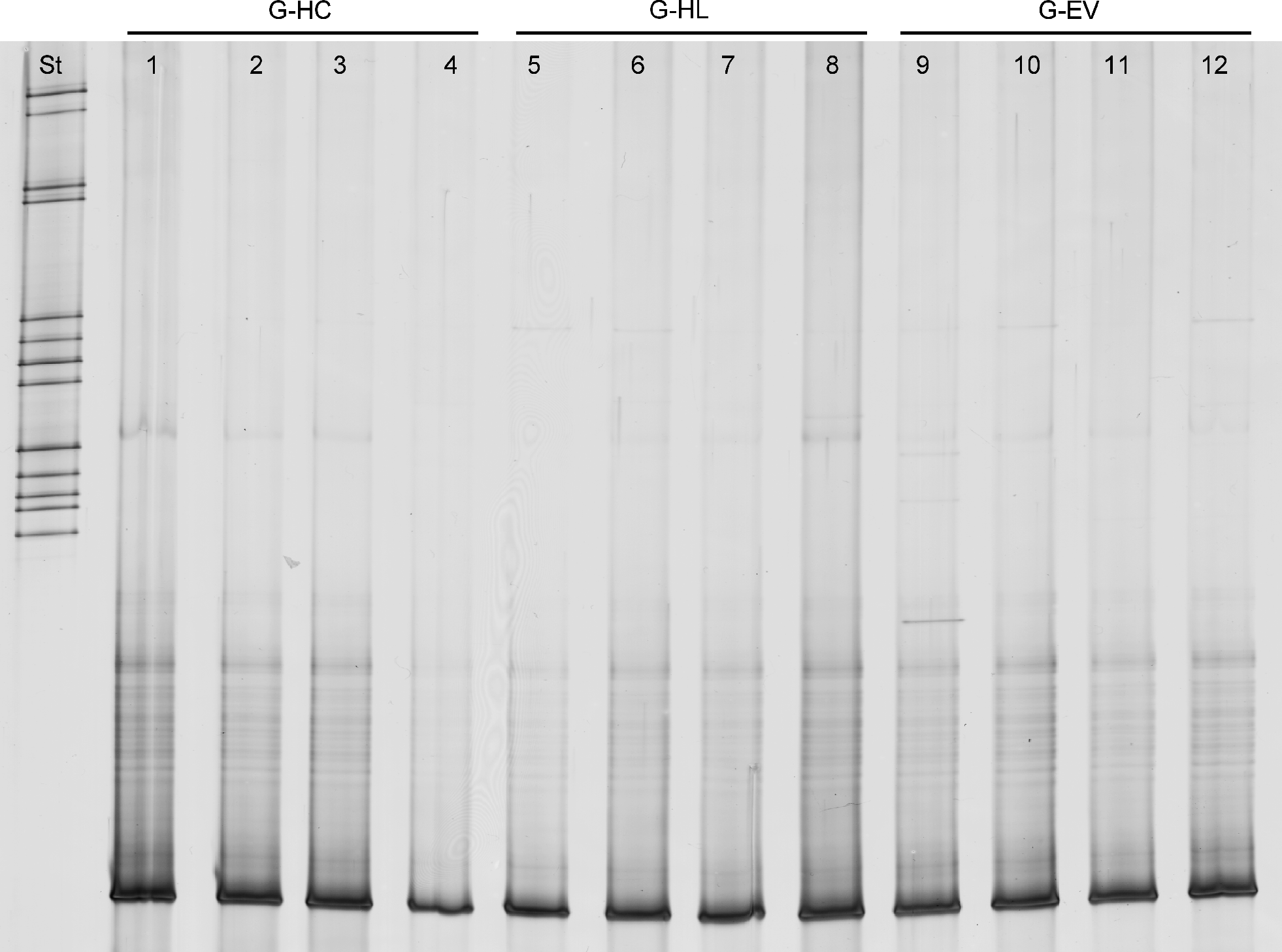

Supplement: Figure S1 — Fungal (18S rRNA gene) DGGE fingerprints obtained from single gut of WCR larvae grown in Haplic Chernozem (G-HC), in Haplic Luvisol (G-HL) and in Eutric Vertisol (G-EV). St: 18S-standard. (TIF) [file pone.0044685.s001.tif]

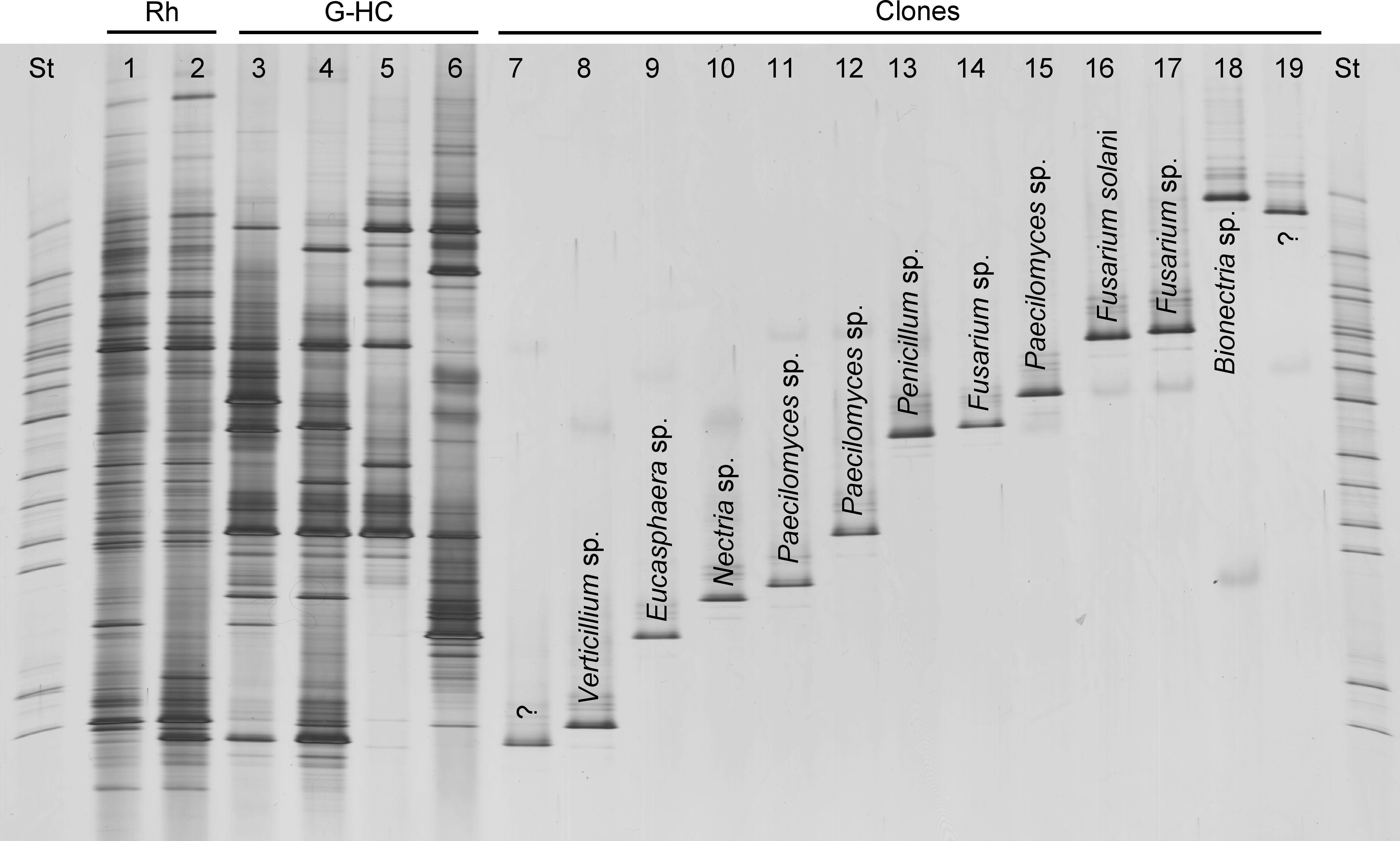

Supplement: Figure S2 — Fungal (ITS) DGGE fingerprints of the fungal communities in the rhizosphere of maize plants grown in Haplic Chernozem (Rh), in single gut samples obtained from WCR larvae feeding on maize plants grown in Haplic Chernozem (G-HC), and DGGE profiles of cloned ITS fragments from single gut samples (clones). Fungi identified by sequencing and blast analysis of cloned ITS fragments are reported above the corresponding DGGE band in the figure. (TIF) [file pone.0044685.s002.tif]
